# Supplementary material for: Faster progression to multiple sclerosis disability is linked to neuronal pathways associated with neurodegeneration: An ethnicity study
Source: PLoS One. 2023 Feb 7;18(2):e0280515. doi: 10.1371/journal.pone.0280515 (PMC9904463; doi:10.1371/journal.pone.0280515)
Supplement: S1 Table — (DOCX) [file pone.0280515.s001.docx]

**Supplementary Table 1. Differentially expressed genes (79 over-expressed and 19 under-expressed) in relapsing-remitting multiple sclerosis patients of Iraqi vs. northern European origin**

| **Gene Symbol** | **Gene title** | **P value** | **False discovery rate** | **Mean**  **Northern Europe** | **Mean**  **Iraq** | **Mean Ratio**  **Northern Europe/Iraq** | **Mean Difference**  **Northern Europe/Iraq** | **Fold Change**  **Northern Europe/Iraq** |
| --- | --- | --- | --- | --- | --- | --- | --- | --- |
| KLHDC10 | Kelch domain containing 10 | <0.0001 | 0.035 | 0.652 | 0.803 | 0.901 | -0.151 | -1.110 |
| GOLGA8A/GOLGA8B/ LOC101930583 | Golgin A8 family, member A/golgin A8 family, member B/uncharacterized LOC101930 | <0.0001 | 0.044 | 1.514 | 1.659 | 0.905 | -0.144 | -1.105 |
| HPGD | Hydroxyprostaglandin dehydrogenase 15-(NAD) | <0.0001 | 0.047 | 0.342 | 0.441 | 0.934 | -0.099 | -1.071 |
| CDC16 | Cell division cycle 16 | <0.0001 | 0.050 | 1.689 | 1.783 | 0.937 | -0.094 | -1.068 |
| CDC16 | Cell division cycle 16 | <0.0001 | 0.054 | 1.182 | 1.273 | 0.939 | -0.090 | -1.065 |
| ABAT | 4-aminobutyrate aminotransferase | <0.0001 | 0.054 | 0.757 | 0.843 | 0.942 | -0.086 | -1.061 |
| MXI1 | MAX interactor 1, dimerization protein | <0.0001 | 0.053 | 1.482 | 1.564 | 0.945 | -0.082 | -1.059 |
| HPGD | Hydroxyprostaglandin dehydrogenase 15-(NAD) | <0.0001 | 0.035 | 0.330 | 0.409 | 0.947 | -0.079 | -1.056 |
| CEP192 | Centrosomal protein 192kda | <0.0001 | 0.054 | 0.858 | 0.933 | 0.949 | -0.075 | -1.053 |
| DLG1 | Discs, large homolog 1 (Drosophila) | <0.0001 | 0.030 | 0.746 | 0.821 | 0.950 | -0.075 | -1.053 |
| MIPEP | Mitochondrial intermediate peptidase | <0.0001 | 0.001 | 0.146 | 0.219 | 0.951 | -0.073 | -1.052 |
| TRIM24 | Tripartite motif containing 24 | <0.0001 | 0.030 | 0.841 | 0.913 | 0.951 | -0.073 | -1.052 |
| UPF3A | UPF3 regulator of nonsense transcripts homolog A (yeast) | <0.0001 | 0.039 | 0.347 | 0.419 | 0.952 | -0.072 | -1.051 |
| SNHG3/SNORA73A | Small nucleolar RNA host gene 3 (non-protein coding)/ small nucleolar RNA, H/ACA box | <0.0001 | 0.040 | 0.244 | 0.315 | 0.952 | -0.071 | -1.050 |
| BEGAIN | Brain-enriched guanylate kinase-associated | <0.0001 | 0.035 | -0.171 | -0.101 | 0.953 | -0.070 | -1.050 |
| PREPL | Prolyl endopeptidase-like | <0.0001 | 0.035 | 0.410 | 0.476 | 0.955 | -0.066 | -1.047 |
| TUBGCP3 | Tubulin, gamma complex associated protein 3 | <0.0001 | 0.033 | 0.206 | 0.272 | 0.956 | -0.066 | -1.047 |
| FADS2 | Fatty acid desaturase 2 | <0.0001 | 0.039 | 0.095 | 0.161 | 0.956 | -0.065 | -1.046 |
| PPP1R12A | Protein phosphatase 1, regulatory subunit 12A | <0.0001 | 0.034 | 1.238 | 1.303 | 0.956 | -0.064 | -1.046 |
| REV1 | REV1, polymerase (DNA directed) | <0.0001 | 0.035 | 1.217 | 1.280 | 0.957 | -0.063 | -1.045 |
| TRIM24 | Tripartite motif containing 24 | <0.0001 | 0.035 | 0.616 | 0.675 | 0.959 | -0.060 | -1.042 |
| DPY19L4 | Dpy-19-like 4 (C. Elegans) | <0.0001 | 0.039 | 0.290 | 0.349 | 0.960 | -0.059 | -1.042 |
| ARHGAP22 | Rho gtpase activating protein 22 | <0.0001 | 0.030 | -0.115 | -0.056 | 0.960 | -0.059 | -1.042 |
| NAA35 | N(alpha)-acetyltransferase 35, natc auxiliary subunit | <0.0001 | 0.041 | 0.340 | 0.398 | 0.961 | -0.058 | -1.041 |
| IREB2 | Iron-responsive element binding protein 2 | <0.0001 | 0.030 | 0.239 | 0.295 | 0.962 | -0.056 | -1.040 |
| LOC100506639/ZNF131 | Uncharacterized LOC100506639/zinc finger protein 131 | <0.0001 | 0.035 | 1.106 | 1.161 | 0.962 | -0.056 | -1.039 |
| SURF1 | Surfeit 1 | <0.0001 | 0.035 | 0.345 | 0.400 | 0.963 | -0.055 | -1.039 |
| TRIT1 | Trna isopentenyltransferase 1 | <0.0001 | 0.035 | 0.982 | 1.037 | 0.963 | -0.054 | -1.038 |
| MIPEP | Mitochondrial intermediate peptidase | <0.0001 | 0.028 | 0.148 | 0.201 | 0.964 | -0.052 | -1.037 |
| KCNG2 | Potassium voltage-gated channel, subfamily G, member 2 | <0.0001 | 0.035 | -0.285 | -0.235 | 0.966 | -0.051 | -1.036 |
| RALGPS1 | Ral GEF with PH domain and SH3 binding motif 1 | <0.0001 | 0.043 | 0.323 | 0.373 | 0.966 | -0.050 | -1.035 |
| CEP70 | Centrosomal protein 70kda | <0.0001 | 0.035 | 0.242 | 0.291 | 0.967 | -0.048 | -1.034 |
| HPGD | Hydroxyprostaglandin dehydrogenase 15-(NAD) | <0.0001 | 0.043 | -0.010 | 0.037 | 0.967 | -0.048 | -1.034 |
| SENP3 | SUMO1/sentrin/SMT3 specific peptidase 3 | <0.0001 | 0.035 | 0.229 | 0.277 | 0.968 | -0.048 | -1.034 |
| ZNF451 | Zinc finger protein 451 | <0.0001 | 0.036 | 1.357 | 1.404 | 0.968 | -0.047 | -1.033 |
| PIN4 | Protein (peptidylprolyl cis/trans isomerase) NIMA-interacting, 4 (parvulin) | <0.0001 | 0.030 | 0.349 | 0.394 | 0.969 | -0.046 | -1.032 |
| KRT81 | Keratin 81 | <0.0001 | 0.041 | -0.007 | 0.038 | 0.969 | -0.046 | -1.032 |
| YTHDC2 | YTH domain containing 2 | <0.0001 | 0.044 | 0.166 | 0.212 | 0.969 | -0.045 | -1.032 |
| WRN | Werner syndrome, recq helicase-like | <0.0001 | 0.035 | 0.389 | 0.434 | 0.970 | -0.045 | -1.031 |
| CEP57 | Centrosomal protein 57kda | <0.0001 | 0.039 | 1.068 | 1.112 | 0.970 | -0.044 | -1.031 |
| PIKFYVE | Phosphoinositide kinase, FYVE finger containing | <0.0001 | 0.044 | 1.614 | 1.658 | 0.970 | -0.044 | -1.031 |
| IRGC | Immunity-related gtpase family, cinema | <0.0001 | 0.030 | -0.207 | -0.163 | 0.970 | -0.044 | -1.031 |
| LDLRAD4 | Low density lipoprotein receptor class A domain containing 4 | <0.0001 | 0.039 | 0.447 | 0.490 | 0.971 | -0.043 | -1.030 |
| CLASP2 | Cytoplasmic linker associated protein 2 | <0.0001 | 0.028 | 0.411 | 0.454 | 0.971 | -0.043 | -1.030 |
| GFPT2 | Glutamine-fructose-6-phosphate transaminase 2 | <0.0001 | 0.028 | -0.165 | -0.122 | 0.971 | -0.042 | -1.030 |
| TIAL1 | TIA1 cytotoxic granule-associated RNA binding protein-like 1 | <0.0001 | 0.043 | 0.673 | 0.714 | 0.972 | -0.041 | -1.029 |
| TRPC1 | Transient receptor potential cation channel, subfamily C, member 1 | <0.0001 | 0.029 | -0.190 | -0.150 | 0.972 | -0.040 | -1.028 |
| TRIM33 | Tripartite motif containing 33 | <0.0001 | 0.036 | 1.443 | 1.483 | 0.973 | -0.040 | -1.028 |
| TP73-AS1 | TP73 antisense RNA 1 | <0.0001 | 0.028 | -0.088 | -0.049 | 0.973 | -0.039 | -1.028 |
| EPM2A | Epilepsy, progressive myoclonus type 2A, Lafora disease (laforin) | <0.0001 | 0.039 | -0.008 | 0.030 | 0.974 | -0.038 | -1.026 |
| AFG3L2 | AFG3-like AAA atpase 2 | <0.0001 | 0.041 | 0.645 | 0.682 | 0.974 | -0.037 | -1.026 |
| CTC-338M12.4 | Uncharacterized LOC101928649 | <0.0001 | 0.041 | 0.184 | 0.221 | 0.975 | -0.037 | -1.026 |
| DFNB31 | Deafness, autosomal recessive 31 | <0.0001 | 0.030 | -0.036 | 0.000 | 0.975 | -0.036 | -1.025 |
| KRIT1 | KRIT1, ankyrin repeat containing | <0.0001 | 0.039 | 0.203 | 0.239 | 0.975 | -0.036 | -1.025 |
| BCAT2 | Branched chain amino-acid transaminase 2, mitochondrial | <0.0001 | 0.050 | 0.036 | 0.072 | 0.975 | -0.036 | -1.025 |
| SLC16A1 | Solute carrier family 16 (monocarboxylate transporter), member 1 | <0.0001 | 0.048 | 0.015 | 0.050 | 0.976 | -0.035 | -1.025 |
| PASK | PAS domain containing serine/threonine kinase | <0.0001 | 0.043 | -0.108 | -0.074 | 0.976 | -0.035 | -1.024 |
| KIAA0586 | Kiaa0586 | <0.0001 | 0.035 | 0.362 | 0.397 | 0.976 | -0.034 | -1.024 |
| TTLL5 | Tubulin tyrosine ligase-like family, member 5 | <0.0001 | 0.041 | 0.118 | 0.150 | 0.978 | -0.032 | -1.023 |
| UPK3B | Uroplakin 3B | <0.0001 | 0.041 | -0.060 | -0.029 | 0.979 | -0.031 | -1.021 |
| NEU3 | Sialidase 3 (membrane sialidase) | <0.0001 | 0.048 | -0.072 | -0.042 | 0.980 | -0.030 | -1.021 |
| RHBDL1 | Rhomboid, veinlet-like 1 (Drosophila) | <0.0001 | 0.044 | -0.228 | -0.198 | 0.980 | -0.030 | -1.021 |
| SPAG8 | Sperm associated antigen 8 | <0.0001 | 0.041 | 0.035 | 0.064 | 0.980 | -0.029 | -1.020 |
| KDM4D | Lysine (K)-specific demethylase 4D | <0.0001 | 0.028 | -0.195 | -0.166 | 0.980 | -0.029 | -1.020 |
| SCARA3 | Scavenger receptor class A, member 3 | <0.0001 | 0.048 | -0.140 | -0.112 | 0.980 | -0.029 | -1.020 |
| MARK1 | MAP/microtubule affinity-regulating kinase 1 | <0.0001 | 0.030 | -0.114 | -0.087 | 0.981 | -0.028 | -1.019 |
| ABCC8 | ATP-binding cassette, sub-family C (CFTR/MRP), member 8 | <0.0001 | 0.041 | -0.088 | -0.061 | 0.981 | -0.027 | -1.019 |
| CAMKK2 | Calcium/calmodulin-dependent protein kinase kinase 2, beta | <0.0001 | 0.030 | -0.094 | -0.067 | 0.982 | -0.027 | -1.019 |
| GLRA1 | Glycine receptor, alpha 1 | <0.0001 | 0.035 | -0.295 | -0.268 | 0.982 | -0.027 | -1.019 |
| RBPMS | RNA binding protein with multiple splicing | <0.0001 | 0.041 | -0.132 | -0.106 | 0.982 | -0.026 | -1.019 |
| OLFM1 | Olfactomedin 1 | <0.0001 | 0.046 | 0.012 | 0.038 | 0.982 | -0.026 | -1.018 |
| DDC | Dopa decarboxylase (aromatic L-amino acid decarboxylase) | <0.0001 | 0.035 | -0.201 | -0.175 | 0.982 | -0.026 | -1.018 |
| CALCA | Calcitonin-related polypeptide alpha | <0.0001 | 0.035 | -0.175 | -0.150 | 0.982 | -0.026 | -1.018 |
| IL1RL1 | Interleukin 1 receptor-like 1 | <0.0001 | 0.028 | -0.156 | -0.131 | 0.983 | -0.025 | -1.017 |
| HEPH | Hephaestin | <0.0001 | 0.041 | -0.182 | -0.158 | 0.984 | -0.023 | -1.016 |
| LAMA2 | Laminin, alpha 2 | <0.0001 | 0.048 | -0.151 | -0.127 | 0.984 | -0.023 | -1.016 |
| OPCML | Opioid binding protein/cell adhesion molecule-like | <0.0001 | 0.035 | -0.233 | -0.210 | 0.984 | -0.023 | -1.016 |
| LAMB4 | Laminin, beta 4 | <0.0001 | 0.035 | -0.212 | -0.192 | 0.986 | -0.021 | -1.015 |
| MCAM | Melanoma cell adhesion molecule | <0.0001 | 0.039 | -0.186 | -0.168 | 0.988 | -0.018 | -1.012 |
| UGT1A1/UGT1A3/UGT1A5/UGT1A8/UGT1A9 | UDP glucuronosyltransferase 1 family, polypeptide A1/UDP glucuronosyltransferase 1 | <0.0001 | 0.043 | -0.216 | -0.235 | 1.014 | 0.020 | 1.014 |
| RIMS1 | Regulating synaptic membrane exocytosis 1 | <0.0001 | 0.030 | -0.116 | -0.136 | 1.014 | 0.020 | 1.014 |
| NCAM2 | Neural cell adhesion molecule 2 | <0.0001 | 0.028 | -0.245 | -0.267 | 1.015 | 0.022 | 1.015 |
| MCF2L2 | MCF.2 cell line derived transforming sequence-like 2 | <0.0001 | 0.039 | -0.121 | -0.143 | 1.016 | 0.022 | 1.016 |
| CRP | C-reactive protein, pentraxin-related | <0.0001 | 0.030 | -0.186 | -0.209 | 1.016 | 0.023 | 1.016 |
| ESRRB | Estrogen-related receptor beta | <0.0001 | 0.035 | -0.206 | -0.233 | 1.018 | 0.026 | 1.018 |
| DGCR14/TSSK2 | Digeorge syndrome critical region gene 14/testis-specific serine kinase 2 | <0.0001 | 0.036 | -0.054 | -0.080 | 1.018 | 0.026 | 1.018 |
| PCDHB1 | Protocadherin beta 1 | <0.0001 | 0.052 | -0.172 | -0.200 | 1.020 | 0.028 | 1.020 |
| POU3F1 | POU class 3 homeobox 1 | <0.0001 | 0.035 | -0.339 | -0.368 | 1.020 | 0.028 | 1.020 |
| IDUA | Iduronidase, alpha-L- | <0.0001 | 0.035 | -0.155 | -0.184 | 1.020 | 0.029 | 1.020 |
| DYRK1A | Dual-specificity tyrosine-(Y)-phosphorylation regulated kinase 1A | <0.0001 | 0.033 | -0.010 | -0.045 | 1.025 | 0.035 | 1.025 |
| YY1 | YY1 transcription factor | <0.0001 | 0.035 | 2.189 | 2.145 | 1.031 | 0.044 | 1.031 |
| TPI1 | Triosephosphate isomerase 1 | <0.0001 | 0.041 | 2.116 | 2.069 | 1.033 | 0.047 | 1.033 |
| EAPP | E2F-associated phosphoprotein | <0.0001 | 0.030 | 1.644 | 1.596 | 1.034 | 0.048 | 1.034 |
| LSM2 | LSM2 homolog, U6 small nuclear RNA associated (S. Cerevisiae) | <0.0001 | 0.035 | 0.963 | 0.906 | 1.040 | 0.056 | 1.040 |
| KLHDC4 | Kelch domain containing 4 | <0.0001 | 0.044 | 0.450 | 0.376 | 1.053 | 0.074 | 1.053 |
| TMEM187 | Transmembrane protein 187 | <0.0001 | 0.035 | 0.365 | 0.264 | 1.072 | 0.101 | 1.072 |
| HLA-J | Major histocompatibility complex, class I, J (pseudogene) | <0.0001 | 0.028 | 2.171 | 2.068 | 1.074 | 0.103 | 1.074 |
| CAMKK2 | Calcium/calmodulin-dependent protein kinase kinase 2, beta | <0.0001 | 0.030 | 1.253 | 0.989 | 1.201 | 0.264 | 1.201 |
