# Supplementary material for: Faster progression to multiple sclerosis disability is linked to neuronal pathways associated with neurodegeneration: An ethnicity study
Source: PLoS One. 2023 Feb 7;18(2):e0280515. doi: 10.1371/journal.pone.0280515 (PMC9904463; doi:10.1371/journal.pone.0280515)
Supplement: S2 Table — (DOCX) [file pone.0280515.s002.docx]

**Supplementary Table 2. Enriched neuronal genes and pathways in relapsing-remitting multiple sclerosis patients with an Iraqi origin**

| **Diseases or function annotation** | **P value** | **Molecules** | **Number of**  **molecules** |
| --- | --- | --- | --- |
| Sensorimotor integration | 0.0013 | IDUA, RIMS1 | 2 |
| Startle response | 0.0031 | DYRK1A, EPM2A, GLRA1, IDUA | 4 |
| Abnormal morphology of tectum mesencephali | 0.0042 | DYRK1A, IREB2 | 2 |
| Branching of neurites | 0.0054 | CAMKK2, CLASP2, DLG1, DYRK1A, NCAM2, NEU3, RIMS1, TRPC1 | 8 |
| Righting reflex | 0.0054 | AFG3L2, GLRA1, PREPL | 3 |
| Delay in initiation of development of oligodendrocytes | 0.0070 | LAMA2 | 1 |
| Proliferation of neocortical neurons | 0.0070 | DYRK1A | 1 |
| Abnormal morphology of dorsal striatum | 0.0070 | IREB2 | 1 |
| Response of olfactory receptor neurons | 0.0070 | CALCA | 1 |
| Hypertrophy of myelin sheath | 0.0070 | POU3F1 | 1 |
| Formation of myelin sheath | 0.0085 | LAMA2, PIKFYVE | 2 |
| Abnormal morphology of Purkinje cells | 0.0102 | EPM2A, IDUA, IREB2 | 3 |
| Accumulation of oligodendrocyte precursor cells | 0.0140 | LAMA2 | 1 |
| Excitation of interneurons | 0.0140 | DLG1 | 1 |
| Quantity of spiny stellate neurons | 0.0140 | RIMS1 | 1 |
| Size of superior colliculus | 0.0140 | DYRK1A | 1 |
| Neurotransmission of thalamocortical axons | 0.0140 | RIMS1 | 1 |
| Endocannabinoid-dependent long-term depression of synapse | 0.0140 | DYRK1A | 1 |
| Abnormal morphology of thalamus | 0.0141 | DYRK1A, IREB2 | 2 |
| Morphology of axons | 0.0156 | CAMKK2, CLASP2, IREB2, POU3F1 | 4 |
| Morphology of white matter | 0.0163 | IREB2, POU3F1 | 2 |
| Myelination | 0.0177 | AFG3L2, DLG1, LAMA2, PIKFYVE, POU3F1 | 5 |
| Binding of neurons | 0.0179 | AFG3L2, CALCA, NCAM2 | 3 |
| Hypertrophy of axons | 0.0209 | POU3F1 | 1 |
| Long-term potentiation of mossy fiber cells | 0.0209 | RIMS1 | 1 |
| Formation of olfactory receptor neurons | 0.0209 | NCAM2 | 1 |
| Cell survival of hippocampal neurons | 0.0209 | PIKFYVE | 1 |
| Abnormal morphology of disorganized barrel cortex | 0.0209 | RIMS1 | 1 |
| Abnormal size of brain | 0.0277 | DYRK1A | 1 |
| Abnormal morphology of somatosensory cortex | 0.0277 | DYRK1A | 1 |
| Sprouting of mossy fibers | 0.0277 | RIMS1 | 1 |
| Myelination of axon bundle | 0.0277 | LAMA2 | 1 |
| Morphology of brain cells | 0.0325 | DYRK1A, EPM2A, IDUA, IREB2 | 4 |
| Paired-pulse facilitation of hippocampal CA1 region | 0.0345 | RIMS1 | 1 |
| Recognition of neurons | 0.0345 | OPCML | 1 |
| Size of presynaptic terminals | 0.0345 | CLASP2 | 1 |
| Abnormal morphology of pyramidal neurons | 0.0379 | DYRK1A, IREB2 | 2 |
| Proliferation of cerebellar granule cell | 0.0413 | CAMKK2 | 1 |
