# Supplementary material for: Faster progression to multiple sclerosis disability is linked to neuronal pathways associated with neurodegeneration: An ethnicity study
Source: PLoS One. 2023 Feb 7;18(2):e0280515. doi: 10.1371/journal.pone.0280515 (PMC9904463; doi:10.1371/journal.pone.0280515)
Supplement: Attachment — Submitted filename: Response to Reviewers 29.11.2022.docx [file pone.0280515.s003.docx]

Prof. Alexander Klistorner

Academic Editor

PLOS ONE	29.11.2022

RE: PONE-D-22-07527

Dear Editor,

Enclosed please find a revised version of our manuscript entitled: Faster progression to multiple sclerosis disability is linked to neuronal pathways associated with neurodegeneration: An ethnicity study.

We have addressed all the points raised during the review process. We hope that the corrected version of our manuscript will be appropriate for publication in PlosOne.

Please find our responses to the Reviewers comments **in bold** as follows:

**A. Reviewer's Responses to Questions**

3. Have the authors made all data underlying the findings in their manuscript fully available?

Reviewer #1: No

Reviewer #2: Yes

**We confirm that all data underlying the findings described in the manuscript are fully available without restriction.**

**Reviewer #1:** The manuscript has been conducted in an admirable methodology as regards to the genetic study, yet there are certain issues that need to be fully elucidated for adopting the proposed generalizability of study findings: The radiologic progression findings for the patients (including but not limited to: MRI demyelinating patch count progression, the black hole count progression, the cerebral volume regression), and the +Ve consanguinity history in the study participants [which is an important possible confounder]. In addition, it has been noted that, in many MS patients of Middle East origin, the incidence of seizures is more as compared to those of Northern European origin.

1. **We thank the Reviewer for the compliment related to the conduction of our study.**
2. **We agree that radiologic progression findings could add information related to disability. However, we used MRI data only for the diagnosis of multiple sclerosis and not as a follow-up variable, and this was related to the fact, as the Reviewer pointed out, that radiologic progression can be defined in more than one way using various MRI variables, and that also spinal cord MRI variables would be of importance to correlate with disability. Therefore, we chose neurologic disability which is systematically performed to be the definer of the disability outcome. As the Reviewer suggested, it will be of importance to analyze in a future study the MRI variables related to disease progression and correlate them with the gene expression findings related to neuronal function. We added this to the paragraph of future consideration in the Discussion.**
3. **There was no consanguinity history in the study participants.**
4. **We did not study the incidence of seizures in the current study.**

**Reviewer #2:** Here, the authors describe a retrospective cohort study in Israel evaluating disability progression in 188 RRMS of Iraqi descent compared to 2,207 MS cases of northern European background. Over the periods of follow-up, a larger proportion of those from Iraq developed moderate disability, this borderline significant (OR=1.35, p=0.049). Similar results were seen evaluating by survival analysis (aHR=1.32, p=0.02). A similar association was seen for risk of developing severe disability was seen but did not reach significance (aHR=1.31, p=0.063). Evaluating PBC gene expression array data, the Iraqi cases showed enriched expression in neuronal pathway genes. The authors conclude this expression data suggests a mechanism underlying a differential disability progression in persons of Iraqi descent acting via increased neurodegeneration.

The fraught international relationships of countries like Iraq with Israel makes the comparison of this population with immigrants from Europe a bit complicated. The nature of people immigrating to Israel from these countries is likely to differ in ways that may be relevant to MS risk, e.g., SES, sociopolitical factors, etc. I think this may merit comment. I am trying to phrase this in a way that is not insensitive or discriminatory and hopefully I have not come across as such. I merely mean to state that because Iraq and other Muslim-majority countries have an acrimonious history with Israel, sometimes including the nations going to war, this may mean that people from Iraq may be differently disposed or able to immigrate to Israel, as compared to those from northern European countries which has not had such issues in their international relations. This has bearing then in the comparability of these patient groups, particularly in the interpretation of observed differences between those of Iraqi and European background as being due to genetic factors, rather than environmental, lifestyle, and other exposures which may differ between the groups. If the authors might please speak to this, I think it relevant.

1. **We thank the Reviewer for his comprehensive review and comments.**

**As all patients with multiple sclerosis included in this study are Jewish, we do not think that the nature of people immigrating to Israel from either Iraq or Northern Europe is likely to differ in ways that may be relevant to MS. Moreover, the immigration to Israel was initiated after the Holocaust in Europe (and not related to the on-going war-situation with the Arab countries) with the establishment of the State of Israel in 1948, and Jewish people immigrated from all over the world regardless to their social, health, or cultural status. We do not think this is relevant to the study from the scientific point of view but rather to the history of Israel.**

**We have added to the Discussion the following comment to emphasize that: the nature of people immigrating to Israel from either Iraq or Northern Europe was not affected by environmental or social variables that could be relevant to MS.**

Further to this, I wonder whether there are immigrants from other countries in the region besides Iraq which might be available for comparison. The authors justify the selection of Iraqis for evaluation because they have observed Iraqi RRMS patients in their clinic to have a worse disease course. It would be supportive to the authors’ interpretation of the results as being something genetically specific to the Iraqis if immigrants from other countries in the region could also be assessed and see whether they also show any differences in disability progression. I appreciate this is a significant amount of new data collection and analysis, however, so if the authors are not able to do this, they could just suggest this as worthy of future investigation.

2. **We agree with the Reviewer that adding new data is currently not feasible and beyond the scope of the study. We have selected the Iraqis for evaluation because we observed they have a worse clinical disease course and therefore we do not see justification to perform future investigation in MS immigrants from other countries in the region besides Iraq.**

I don’t see how enriched expression of genes involved in neuronal function, including neuronal proliferation and structure and myelin sheath, is indicative of a pathway of increased neurodegeneration. It becomes apparent in the body text that proliferation and morphology loci were downregulated in the Iraqi patients, while myelination and myelin repair and synapse loci were upregulated. While the neuronal proliferation downregulation might be a negative, I am uncertain that the morphology downregulation is clearly deleterious. Also, myelination and myelin repair loci being upregulated would seem a good thing. This all of course presumes that the expression seen in PBMCs is relevant to expression that may be occurring in the CNS. I think these expression data are suggestive, albeit a bit mixed, and so the results should be framed a bit more conservatively, as they don’t clearly indicate a neurodegeneration profile and in any case the extrapolation of these results to long-term disability progression is uncertain.

3. **We agree with the Reviewer that the expression** **data are suggestive as they are seen in PBMCs and therefore the relevance to the CNS is not direct. Nevertheless, the relatively large number of genes in the enriched expression analysis merits inspection of the findings. We intend in a future study to investigate the MRI findings of these patients in relevance with the gene expression findings to evaluate whether Iraqi -origin MS patients will demonstrate MRI markers of neuronal loss and brain atrophy as compared to the North-European originated patients.**

**We added this paragraph to a future consideration paragraph in the Discussion.**

Further to this, please acknowledge the limitation in the Discussion that expression in PBMCs may not be reflective of the expression profile in the CNS. This goes without saying but the authors are framing results for expression of genes involved in CNS function and repair in PBMCs, so it would be good to be explicit and acknowledge this limitation and the need to assess whether such differences in expression are also seen in the CNS.

4. **Added in the Discussion**

Please consider evaluating disability as a continuous term and looking at rate of change. Also, please consider looking at time to event for reaching EDSS 4 and 6, as these are more typical cutpoints for disability than moderate and severe. This would better support the framing of conclusions as Iraqis having faster disability progression.

5. **We performed survival analysis looking at the rate of EDSS progression over-time as is shown in Fig. 1. As we had a long-term data, we chose to show disability progression over a period of 15 years. At each time point it is easy to identify the percent of patients that reached EDSS 3 or EDSS 6. The cutpoints of EDSS=3 and EDSS=6 are the frequent EDSS scores used to define moderate and severe disability in MS clinical trials, and therefore are preferred than EDSS4 which already signifies decreased walking distance.**

The framing of results as Iraqi-descent patients having a greater risk of moderate but not severe disability is not really appropriate. The magnitudes of association are statistically identical and the level of statistical significance is not that different either. Just because one falls below p=0.05 but not the other is not reason to disregard the association for severe disability. The moderate disability association can certainly be framed as having a higher level of statistical evidence but it’s not right to say there was no association for severe disability.

6. **We agree with the Reviewer that from the statistical perspective the magnitudes of association was similar. As the 'N' in each EDSS category was limited we could not demonstrate a rate of change with adequate statistical power when using continuous variable for severe disability. For these reasons and mostly because of the clinical consideration, a dichotomy was used, calculation the odds ratio to reach moderate or progressive disability, showing that an Iraqi origin was associated with increased risk of progression to moderate disability (adjusted hazard ratio 1.323; 95% CI, 1.049-1.668, p=0.02) but not to severe disability defined as EDSS score >=6.0 (i.e., walking aids are required for a distance of 100 meters, (adjusted hazard ratio 1.311; 95% CI, 0.918-1.874, p=0.063).**

The final paragraph of the Discussion states that there is an overexpression of genes involved in repressing neuronal proliferation and downexpression of genes involved in myelination**.** This conflicts with results presented earlier, however, where it says that genes involved in neuronal proliferation were downregulated and that those for myelination and myelin repair were upregulated. Please assess and remedy any discrepancy.

7. **Please note that in the final paragraph it is stated that ….”** over-expression of genes associated with **inhibition** of neuronal proliferation…..”, **and therefore, there is no conflict with the results presented earlier.**

8. Abstract:

- Please state where the study was conducted. **added**

- Please specify that the 2,207 cases are also RRMS. **specified**

- Please specify how disability was assessed and what defines moderate and severe disability. **Specified and defined.**

- Please specify what models are adjusted for. Also, for internal comparability, please specify what the association by logistic regression adjusting for these factors is.

**We specified in the Methods and results and Tables 3 and 4 that the logistic regression was adjusted for age at onset, gender. disease duration, and immunomodulatory treatment.**

Furthermore, as is shown in the Results we also calculated the HR for progression to moderate and severe disability and found that the risk for moderate disability progression was significantly higher (hazard ratio, 1.323; 95% confidence interval, 1.049 to 1.668; p=0.0183) for relapsing-remitting MS patients of Iraqi origin as compared to North European, while the risk for severe disability did not reach statistical significance (hazard ratio, 1.311; 95% confidence interval, 0.918 to 1.874; p=0.136).

- Please specify that the gene expression enrichment was different for Iraqi vs European cases and consider specify the numbers of upregulated and downregulated genes.

**Specified and the numbers of upregulated and downregulated genes and the numbers of patients evaluated were added in the Abstract.**

9. Introduction:

- Consider different word choice for “the Orient”. **We have deleted the word.**

10. Methods:

- Please specify whether the patients from European countries are also Jewish? It is stated that the Iraqis are so I reckon this is relevant. **We clarified that all patients in the study are Jewish.**

- In the inclusion criteria, it is stated that Iraqi-descent patients were included but earlier in the Methods it states they were Jewish. Please ensure consistency. **As specified in the response to the previous comment all patients in the study are Jewish with either Iraqi origin, or Northern Europe (Ashkenazi) origin.**

- Further to this, the inclusion criteria states that the European patients had to be Ashkenazi Jews. Were the Iraqis also Ashkenazi or were they a mixture of Jewish types**? Iraqis by definition cannot be Ashkenazi.** **Iraqi originated Jews are non-Ashkenazi (defined as Sephardic or Orientals) and Northern Europe origin Jews are defined as Ashkenazi. Mixed patients (one parent from Northern Europe, one parent from Iraq) were not included in the study. We added this to the Methods section to clarify.**

Is this likely to have relevance for analyses? If so, please speak to this in the Discussion. The authors might also consider, sample allowing, to do a sensitivity analysis constraining the Iraqis to Ashkenazi Jews and seeing whether disability progression differs between them and the European group. **Please see the response to the 2 previous comments – there is no relevance to the analyses as these are by definition 2 distinct groups.**

- Were analyses adjusted for DMTs considered? If so and results did not materially differ, this can just be stated.

**Added in the Methods and the Results.**

Formatting/other:

- Please use sex and male/female terminology, rather than gender and men/women. **Corrected.**
